# TRPV4 subserves physiological and pathological elevations in intraocular pressure

**DOI:** 10.21203/rs.3.rs-4714050/v1

**Published:** 2024-07-12

**Authors:** Sarah N. Redmon, Monika Lakk, Yun-Ting Tseng, Cristopher N. Rudzitis, Jordan E. Searle, Feryan Ahmed, Andrea Unser, Teresa Borrás, Karen Torrejon, David Krizaj

**Affiliations:** University of Utah; University of Utah; University of Utah; University of Utah; University of Utah; Humonix; Humonix; University of North Carolina; Humonix; University of Utah

**Keywords:** TRPV4, glaucoma, trabecular meshwork, outflow facility, intraocular pressure

## Abstract

Ocular hypertension (OHT) caused by mechanical stress and chronic glucocorticoid exposure reduces the hydraulic permeability of the conventional outflow pathway. It increases the risk for irreversible vision loss, yet healthy individuals experience nightly intraocular pressure (IOP) elevations without adverse lifetime effects. It is not known which pressure sensors regulate physiological vs. pathological OHT nor how they impact the permeability of the principal drainage pathway through the trabecular meshwork (TM). We report that OHT induced by the circadian rhythm, occlusion of the iridocorneal angle and glucocorticoids requires activation of TRPV4, a stretch-activated cation channel. Wild-type mice responded to nocturnal topical administration of the agonist GSK1016790A with IOP lowering, while intracameral injection of the agonist elevated diurnal IOP. Microinjection of TRPV4 antagonists HC067047 and GSK2193874 lowered IOP during the nocturnal OHT phase and in hypertensive eyes treated with steroids or injection of polystyrene microbeads. Conventional outflow-specific *Trpv4* knockdown induced partial IOP lowering in mice with occluded iridocorneal angle and protected retinal neurons from pressure injury. Indicating a central role for TRPV4-dependent mechanosensing in trabecular outflow, HC067047 doubled the outflow facility in TM-populated steroid-treated 3D nanoscaffolds. Tonic TRPV4 signaling thus represents a fundamental property of TM biology as a driver of increased *in vitro* and *in vivo* outflow resistance. The TRPV4-dependence of OHT under conditions that mimic primary and secondary glaucomas could be explored as a novel target for glaucoma treatments.

## INTRODUCTION

Glaucoma, a leading cause of irreversible blinding disease that affects more than 80 million people across the world (1), is characterized by unique pathologies in tissues that regulate the production and drainage of aqueous humor in the anterior eye and the retina (2, 3). Most cases of glaucoma are polygenic and heritable, with genome-wide association studies recognizing hundreds of disease risk loci in individuals with European, Asian and African ancestries (4, 5). Risk factors such as advanced age, sex, myopia, and intraocular pressure (IOP) contribute to the etiology and progression of glaucoma, with the importance of the biomechanical milieu underscored by strong correlations between the extent and duration of ocular hypertension (OHT), rate of glaucoma progression, and its severity (6, 7). Given the current limitation of clinical IOP lowering interventions, a better understanding and targeting of mechanosensitive pathways within tissues that produce and drain aqueous humor but fail in glaucoma would benefit treatment.

The trabecular meshwork (TM), a circumocular tissue composed of extracellular matrix (ECM) beams populated by pressure-sensing contractile smooth muscle-like cells, is the principal regulator of IOP homeostasis and an effector of pressure-induced remodeling in glaucoma (8, 9). An increase in IOP distends ECM beams, modulates the expression of hundreds of TM genes, drives ECM secretion, and induces fibrotic remodeling that impedes the passage of aqueous humor into the canal of Schlemm (10, 11). Compelling evidence indicates that OHT maintenance requires tonic stimulation of pressure sensors upstream from TM Rho signaling, cytoskeletal dynamics and remodeling of the juxtacanalicular extracellular matrix (JCT ECM). TM responsiveness to mechanical stress, elevated TGFb concentrations and chronic steroid treatment converge at the level of increased ECM stiffness and actomyosin contractility as Rho kinase (ROCK)-dependent increases in JCT resistance to fluid flow (10, 12, 13). This process has been associated with upregulation of the mTOR-AKT1 pathway, SMAD2/3 transcription, autophagy (14), overactivation of integrin-based cell-ECM contacts (15), release of matrix metalloproteinases (MMPs) (16), and altered expression of TM proteins related to aging, DNA structure, cytochrome P450 signaling and cell differentiation (17) while the identity of pressure sensing molecules that drive these remodeling pathways remains poorly understood. Cultured human TM cells are highly mechanosensitive, responding to physiological (5–15 mm Hg) pressure steps with Na^+^, K^+^ and Ca^2+^ currents mediated by Piezo1, TRPV4 and TREK-1 channels (18–21). Piezo1 and TREK-1 activity were proposed to respectively increase and decrease the outflow facility (18, 20, 21) whereas the functional role of TRPV4 channels, which show prominent TM expression in rodents and humans, remains under debate (22–25).

TRPV4 (Transient Receptor Potential Vanilloid Isoform 4) is a polymodal nonselective cation channel that can be activated by mechanical stressors (tension, shear, compression, swelling), temperature, metabolites of phospholipase A2, UV-B photons and the selective synthetic agonist GSK1016790A (GSK101) (26–28). Worms with mutations in the TRPV4 homolog osm-9 lose responsiveness to mechanical stimuli (29), *Trpv4* knockout mice are deficient in systemic and cellular responsiveness to osmotic and mechanical stress (29–31) and gain- and loss-of-function TRPV4 mutations cause retinal degeneration (32), sensory/motor neuropathies, and skeletal dysplasias (33, 34). TRPV4 channels regulate the permeability of epithelial and endothelial layers (35–37) and function of fluid-regulating tissues and organs such as heart, kidney, bladder, lung and eye (38, 39), with modulation of TRPV4 activity shown to modulate intracranial pressure (40), blood pressure (41), bladder pressure (42), alveolar pressure (43), and systemic osmoregulation (44). TRPV4 channels mediate the majority of the pressure-evoked inward current and attendant [Ca^2+^]_i_ elevations in TM cells (23) yet current hypotheses disagree about their function in mechanosensing, regulation of outflow resistance and IOP homeostasis: TRPV4 activation has been implicated in IOP lowering and elevation, and associated to downstream phosphoinositide signaling (22), polyunsaturated fatty acid metabolism and release (23, 25), cytoskeletal and focal adhesion dynamics (23, 45) and eNOS activation (22, 24). Genetic studies using global knockout animals yielded conflicting conclusions regarding the TRPV4-dependence of IOP (23–25) and it remains unclear whether the data reflect activation of different TRPV4 pools within the anterior eye or context-dependence of TRPV4 signaling studied under different OHT paradigms (27, 39). The objective of this study was to combine pharmacological and genetic strategies to clarify the role of TRPV4 activity in normotension and OHT, assess its contribution to the circadian IOP rhythm and ascertain its role as a modulator of outflow facility in an *in vitro* model of steroid-induced increase in outflow resistance. Our results show that TRPV4 plays an obligatory role in pressure- and steroid-induced OHT and in the maintenance of nocturnal IOP.

## MATERIALS AND METHODS

### Study Design

This study tests the hypotheses that TRPV4 channels contribute to IOP homeostasis under ocular normotensive and hypertensive conditions. Multiple IOP conditions (diurnal, nocturnal, angle-occlusion, chronic steroid exposure) and drug administration protocols (topical, intracameral delivery) were combined with genetic models to define to contribution of TRPV4 activity to steady-state IOP levels. TRPV4-dependence of trabecular outflow was further assessed in a 3D-nanoscaffold system in the absence of inflow, uveoscleral and Schlemm’s canal components of flow regulation. The sample sizes were determined based on previous studies. Animals in agonist/antagonist and PBS groups were randomized and evaluated by staff blinded to treatments, with male and female animals pooled due to identical responses to pharmacological and genetic interventions. IOP measurements were noninvasive. RGC counts were conducted in retinas isolated from enucleated eyes.

### Animals.

Animal handling, anesthetic procedures and experiments were performed in accordance with theNIH Guide for the Care and Use of Laboratory Animals and the ARVO Statement for the Use of Animals in Ophthalmic and Vision Research and were approved by the Institutional Animal Care and Use Committees at the University of Utah. Mouse strains were C57BL/6J (JAX, Bar Harbor, ME), global *Trpv4*^−/−^ nulls with excised exon-encoding transmembrane domains 5 and 6 (developed by Dr. Wolfgang Liedtke; (124)), *Trpv4*^*fl/fl*−^ mice were developed at the University of Utah from ES clones obtained from the KOMP repository *Trpv4*^*trn1a(KO<P)Wtsi*^ (55). The lacZ reporter was removed by crossing *Trpv4*^*trn1a(KO<P)Wtsi*^ chimeras with mice expressing flippase. Subsequent mating with *Mgp* (Matrix-Gla protein) Cre mice (54) yielded animals lacking functional TRPV4 expression in the conventional outflow pathway. Flox and Cre genotypes were confirmed by PCR from tail snips using the primers: TRPV4-ttR: GTCCTCATACCATGTGAGCTGAACC, TRPV4-F: TCCCTGTGTCACTTTCTACACCTGG, MGP-Cre WT F: TGCCTACGAGATCAACAGAG, MGP-Cre WT R: ATGTGGTTACACCTCCACAC, MGP-Cre Mut F: CTGGAGTTTCAATACCGGAG, and MGP-Cre Mut R: ATGTGGTTACACCTCCACAC. PCR was performed using the following protocol: *Trpv4*^*flx*^: 94°C 5 min, [94°C 30s, 65°C 30s decrease 1°C/cycle, 72°C 40s,]x10, [94°C 30s, 55°C 30s, 72°C 40s]x30, 72°C 5 min. *Mgp*^*Cre*^: 94°C 2 min, [94°C 30s, 58°C 30s, 72°C 1min]x40, 72°C 2 min. Samples were held at 4°C until run on a 2% agarose gel. Expected product sizes were 527, 666, 467, and 690 for the WT, TRPV4^fl^, Cre^−^ and Cre^+^ alleles, respectively. Animals were maintained in a pathogen-free facility with a 12-hour light/dark cycle, temperature set at ~22–23°C and *ad libitum* access to food and water.

### Steroid-induced mouse glaucoma model.

Mice were administered daily topical 0.1% DEX sodium phosphate ophthalmic eyedrops (Bausch & Lomb Inc.) three times a day (10–11 AM, 2–3 PM, 6–7 PM) for ~12 weeks. An age-matched cohort was treated with sterile PBS drops. Mice were gently restrained for ~30 seconds after drop application to prevent grooming and allow for glucocorticoid permeation.

### mTM/pTM purification and culture.

Mouse eyes were enucleated and rinsed in PBS at RT and isolated as described as described (19, 20, 72). The TM tissue was rinsed in PBS containing 1% AA (ThermoFisher) and digested (1% AA, 4 mg/mL collagenase A (MilliporeSigma), 4 mg/mL BSA in PBS) for 2 hours at 37°C. Following a rinse (DMEM, GIBCO supplemented with 10% FBS and 1% AA) the cells were dissociated, strained through a 70 μm filter (Miltenyi Biotech), transferred to 25 cm^2^ flasks and incubated at 37°C for 24 hours.

Primary human TM (pTM) cells were cultured from human donor eyes obtained from the Lions Eye Bank as described (19, 20, 72). Experiments were conducted with P1-P4 cells.

#### Reagents.

The TRPV4 agonist GSK1016790A (GSK101) and antagonists HC-067047 (HC-06) and GSK2193874 (GSK219) were purchased from Cayman Chemical. GSK101 (1 mM), HC-06 (20 mM) and GSK219 (10 mM) DMSO stocks were diluted in extracellular saline, with the final DMSO concentrations not exceeding 0.1%. Salts were purchased from Sigma or VWR.

#### Reverse transcription and semi-quantitative Real-Time PCR.

mTM/pTM cells were used for mRNA expression analyses (45, 71). Total RNA was isolated using the Arcturus RNeasy Plus Micro Kit (Qiagen) with 500 ng of total RNA used for reverse transcription. First-strand cDNA synthesis and PCR amplification of cDNA was performed using qScript^™^ Ultra SuperMix cDNA synthesis kit. SYBR Green based real-time PCR was performed using Apex qPCR Master Mix (Genesee Scientific). The results were performed in triplicate of at least three to four experiments. The comparative CT method (ΔΔC_T_) was used to measure relative gene expression where the fold enrichment was calculated as: 2^-[ΔCT (sample)−ΔCT (calibrator)]^ after normalization. *Gapdh* was utilized to normalize the fluorescence signals. Primer sequences are shown in Table 1.

#### Cryosection and wholemount immunohistochemistry.

Mice were euthanized, and eyes fixed in 4% PFA for one hour at room temperature (RT) as described (31, 123). Tissue was cryoprotected in 15% sucrose and 30% sucrose for 45 min at room temperature and overnight at 4°C, respectively, and the eyes embedded in OCT (Tissue-Tek). 12–16 μm slices were cut with a cryostat, mounted on Superfrost plus slides (Thermofisher), dried and kept at −80°C. Wholemount retinas were dissected out, placed in 1X PBS and kept at 4°C. For staining, cryosections and wholemounts were rinsed in PBS (3 × 5 min) and blocked in 5% FBS and 0.3% Triton-X in 1X PBS for 30 min (sections) and 60 min (retinas) at RT. Primary antibodies (mouse aSMA 1:500; Abcam; rabbit TRPV4 1:1000 Lifespan BioSciences; rabbit or guinea pig RBPMS, 1:1000 PhosphoSolutions) were prepared in 2% BSA and 0.2% Triton-X in 1X PBS. Tissue was incubated overnight (cryosections) or 3 days (wholemounts) at 4°C. Slices/wholemounts were rinsed 3 times for ~5 minutes in 1X PBS, incubated with secondary antibodies for 1hr at RT and washed 3x for 5 min in 1X PBS. Secondary antibodies were goat anti-mouse Alexa Fluor 594 1:1000 Invitrogen; goat anti-rabbit AlexaFluor 488 or 594 1:1000 Invitrogen/ThermoFisher Scientific; goat anti-guinea pig Cy3 1:1000 Jackson ImmunoResearch). Cryosections were counterstained (DAPI-Fluoromount-G, Southern Biotech) and coverslipped. Wholemounts were flattened by making 4 cuts around the perimeter of the retina before applying the mounting solution. Immunofluorescence was acquired with confocal microscopes (Olympus FV1200, 20x water; Zeiss LSM710, 40x oil). For retinal wholemounts, a z-stack was collected in four quadrants of the peripheral retina and RBPMS^+^ cells manually counted using ImageJ (NIH).

#### Microbead and pharmacological compound injections.

Mice were anesthetized with an intraperitoneal (IP) injection of ketamine/xylazine (90 mg/10 mg / kg of body weight) and ocular sensations numbed with 0.5% proparacaine hydrochloride applied with 1% tropicamide ophthalmic solution (Bausch & Lomb). IOP was elevated unilaterally as in Ryskamp et al. (2016). A guide hole was made in the cornea with a 30-gauge needle, cornea was depressed to displace AH. Followed by microinjection of 2 μl of 10 μm polystyrene microbeads (MBs; FluoSpheres; Bangs Laboratories) into the anterior chamber over 60 sec. A bubble was applied to seal the cornea. Intracameral injection was performed as described above with 2 μL of GSK101 (1 μM) or HC-06 (100 μM). Contralateral eyes were injected with PBS (for MB-injected eyes) and PBS + DMSO vehicle (for pharmacological testing).

#### Cryosection and wholemount immunohistochemistry.

The tissues were prepared as described (31, 123). Primary antibodies (mouse a-SMA 1:500; Abcam; rabbit TRPV4 1:1000 Lifespan BioSciences; rabbit or guinea pig RBPMS, 1:1000 PhosphoSolutions) were prepared in 2% BSA and 0.2% Triton-X in 1X PBS. Immunofluorescence was acquired with confocal microscopes (Olympus FV1200, 20x water; and Zeiss LSM710, 40x oil). For retinal wholemounts, a z-stack was collected in four quadrants of the peripheral retina and RBPMS^+^ cells manually counted using ImageJ.

#### Calcium imaging.

mTM cells were loaded with 5 μM Fura-2-AM for 30–45 minutes and perfused with isotonic saline (pH 7.4) containing (in mM): 98.5 NaCl, 5 KCl, 3 MgCl_2_, 2 CaCl_2_, 10 HEPES, 10 D-glucose, 93 mannitol. Epifluorescence imaging was performed as described (23, 45) using inverted Nikon Ti or upright Nikon E600 FN microscopes with 20x (0.75 N.A. oil) and 40x (1.3 N.A. oil & 0.8 N.A. water) objectives and Nikon Elements software. Results represent averages across cells obtained from 2–4 slides from 2–3 independent experiments.

### Outflow Facility using Humonix Biosciences 3D-HTM^™^

#### Biomimetic Scaffold Fabrication:

SU-8 2010 (MicroChem Corp., Newton, MA) was used to develop free-standing biomimetic porous microstructures that served as scaffolds on which primary HTM cells were cultured. Scaffolds were fabricated using standard photolithographic techniques as previously described (58). Briefly, a release layer was spin-coated on the wafer and baked at 150°C. SU-8 2010 was applied by spin-coating to final thickness of 5 mm, then baked at 95°C and cooled to room temperature. The resist was UV-exposed through a mask containing the desired pattern, baked at 95°C and developed in PGMEA developer (MicroChem Corp.) SU-8 scaffolds with the desired features were released from the substrate, washed with acetone, and sterilized using 70% ethanol. After the ethanol dried, the scaffolds were placed under UV light for 1 h per side and then coated with 1% gelatin on both sides and allowed to dry.

#### 3D-HTM tissue constructs and treatments:

Methods to create the 3D tissue of the TM were described in Torrejon et al. (2016). Briefly, primary TM cells were isolated from two healthy donor tissue rings discarded after penetrating keratoplasty (Cell Applications, San Diego, CA) and plated in 1% gelatin-coated 75 cm2 cell culture flasks and cultured in modified Improved Minimum Essential Medium (IMEM; ThermoFisher Scientific) containing 10% premium select heat-inactivated FBS (Atlanta Biologicals, Lawrenceville, GA) and 0.1 mg/mL gentamicin (ThermoFisher Scientific). Fresh culture medium was supplied every 48 hours, and cells were maintained in a humidified atmosphere with 5% carbon dioxide until confluence. pTM cells were then trypsinized using 0.25% Trypsin/0.5 mM EDTA (Thermo Fisher Scientific) and subcultured onto 1% gelatin-coated microporous scaffolds attached to aluminum rings and placed in 24-well plates. The cells were seeded at a density of 40,000–50,000 cells per scaffold. Once confluent (12–14 days in culture), the pTM constructs were serum starved (1% FBS–IMEM) for 24 hrs before treatment with vehicle or 9-prednisolone acetate (9-PA; 300 nM) for 3 days. On day 3, samples were treated with vehicle or 9-PA ± 1 μM HC-06 for 6 days followed by perfusion studies. The 3D-HTM constructs were perfused with high glucose (4.5 g/l) DMEM (Thermo Fisher Scientific) supplemented with gentamicin that contained vehicle, 9-PA ± 1μM HC-06. Perfusion medium with treatments flowed from the apical-to-basal direction across the TM cells. The temperature was maintained at 34°C throughout the experiment with pressure continuously monitored and recorded. After perfusion, the hydraulic conductivity (outflow facility) of the constructs was calculated from the inverse of the slope of the pressure versus flow per unit surface area.

### Statistical Analysis

Group sizes were determined based on preliminary experiments, with data analyzed by GraphPad Prism 10.0. Unless specified otherwise, an unpaired t-test was used to compare to means. One-way or two-way ANOVA with Tukey post-hoc comparisons was used to compare three or more means. Data are presented as mean ± S.E.M.

## RESULTS

### Microinjection of the TRPV4 agonist lowers and topical administration elevates IOP

**Figure 1:**
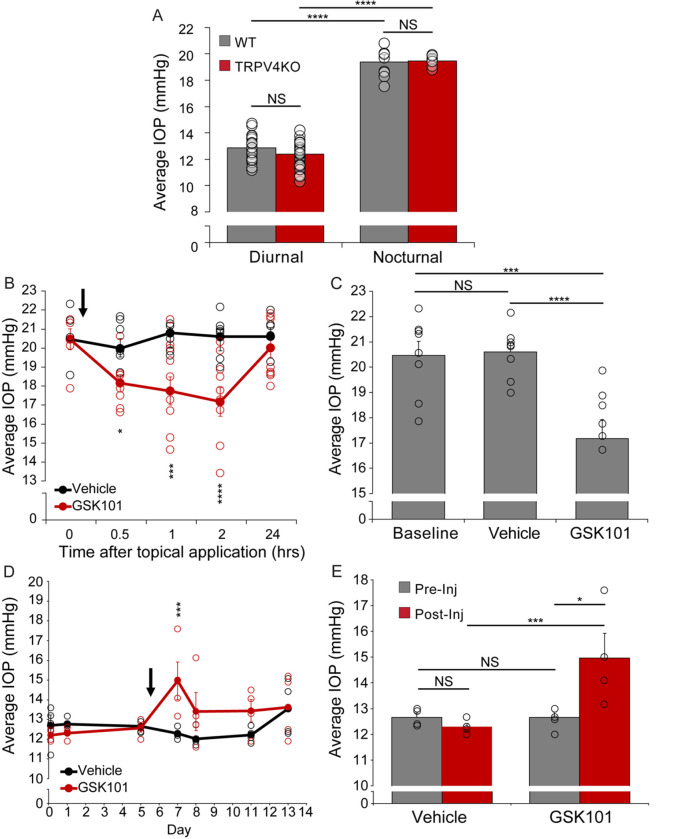
TRPV4 activation and inhibition modulate circadian changes in IOP. (**A**) Average IOP in WT (gray bars) and pan *Trpv4*^−/−^ (red bars) mice collected either diurnally (WT: N = 25 mice; *Trpv4*^−/−^: N = 50 mice) or nocturnally (WT: N = 9 mice; *Trpv4*^−/−^: N = 8 mice). Error bars represent mean ± SEM. NS = not significant; ****P < 0.0001; two-way ANOVA with Tukey post-hoc comparison tests. Ablation of the *Trpv4* gene is not associated with altered IOP under diurnal and nocturnal conditions. (**B**) Topical administration (black arrow) of 20 μM GSK101 (red trace) or 0.01% DMSO in PBS (vehicle; black trace) during the nocturnal phase. GSK101 induced IOP lowering that lasted for ~2 hours. Error bars, mean ± SEM (N = 8 eyes per group from 8 mice; each mouse was treated with GSK101 in one eye and vehicle in the contralateral eye). IOP was measured 0.5, 1, 2, and 24 hours after eyedrop application. Statistical comparison was between vehicle and GSK101 at each timepoint (*P < 0.05, ***P < 0.001, ****P < 0.0001; Two-way ANOVA with Tukey post-hoc comparisons). (**C**) Data summary from B of the average IOP 2 hrs after topical application of 20 μM GSK101 compared to the pre-injection IOP baseline. (**D**) Average IOP of eyes injected with either 1 μM GSK101 or 0.01% DMSO/PBS vehicle in the anterior chamber of WT mice during the day (black arrow). IOP was measured 24, 48, 5 and 7 days after the injection. Error bars represent mean ± SEM (N = 4 eyes per group from 4 mice; each mouse was injected with GSK101 in one eye and vehicle in the contralateral eye). Statistical comparison was between vehicle and GSK101 at each time point; non-significant relationships are not shown. (***P < 0.001; Two-way ANOVA with Šidák post-hoc comparisons. (**E**) Data summary for the 24 hr post injection time point shows significant increase in IOP in GSK101-but not PBS-stimulated eyes. Error bars represent mean ± SEM. NS = non-significant, * P < 0.05, *** P < 0.001, ****P< 0.0001.

The polymodal cation channel TRPV4 has been suggested to set the hypotensive phase of the circadian IOP clock (24) and induce hypertension in response to mechanical stress (23). We employed tonometry to address whether the results might correspond to differential experimental protocols. Consistent with previous reports (24, 46, 47), nocturnal IOP in wild-type C57BL/6J mice was higher compared to diurnal levels (Fig. 1A). Topical administration of the agonist GSK1016790A (GSK101) lowered nocturnal OHT while animals treated with the DMSO/PBS vehicle did not exhibit IOP changes (Fig. 1B and C). In contrast to the hypotensive effect of topical administration, intracameral GSK101 microinjection elevated diurnal (Fig. 1D and E). Tissue permeability and anterior chamber access of TRPV4 modulators should therefore be considered relevant stimulation parameters.

**Figure 2:**
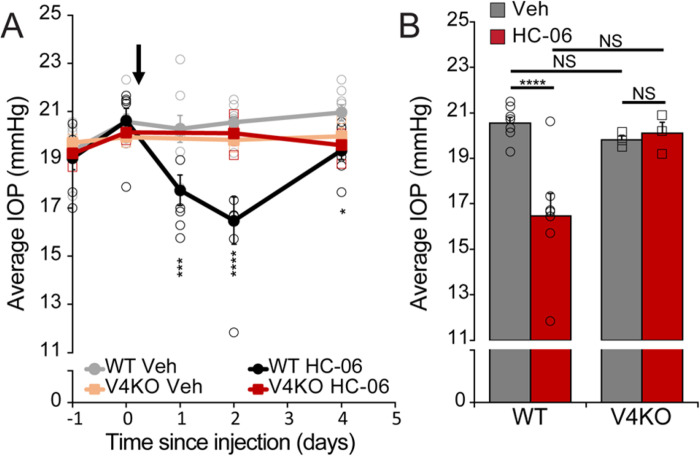
HC-06 injection lowers nocturnal IOP in WT but not *Trpv4*^−/−^ mice. (**A**) Time course of average IOP in WT and V4KO eyes injected with vehicle (WT: N = 8 eyes *Trpv4*^−/−^: N = 3 eyes) or HC-06 (WT: N = 7 eyes; *Trpv4*^−/−^: N = 3 eyes). WT and *Trpv4*^−/−^ mice were injected with HC-06 in one eye and DMSO/PBS vehicle in the contralateral eye. One eye was excluded from analysis for post-injection complications. Nocturnal IOP was measured 24, 48, and 96 hours after injection. Error bars represent mean ± SEM. Asterisks represent statistical differences comparing WT + HC-06 vs WT + Veh. Other group comparisons are not significant and therefore not shown (two-way ANOVA with Tukey multiple comparisons). *P <0.05, ***P<0.001, ****P<0.0001. (**B**) Data summary from A at 48 hours after injection shows that HC-06 lowers nocturnal increases in intraocular pressure in WT mice, but not in TRPV4^−/−^ mice. The data represents mean ± SEM. NS = non-significant, ****P < 0.0001.

Pan-*Trpv4*^−/−^ mice maintained the diurnal to nocturnal phase difference as seen in wild-type animals. There was no discernable difference in intraocular pressure between WT and *panTrpv4*^−/−^ mice in the diurnal or nocturnal phase (Fig. 1A). While IOP-independence of TRPV4 ablation could be interpreted as an argument against its involvement in circadian regulation, injection of the antagonist HC067047 (HC-06) which binds the S1-S4 pore domain of the protein(48) lowered nocturnal IOP (Fig. 2A and B). Injection of GSK2193874 (GSK219), a structurally dissimilar inhibitor of TRPV4, likewise reduced IOP, an effect not statistically different from the impact of HC-06 (Fig. S2). Given that HC-06 microinjections into the nocturnal-phase eyes of *Trpv4*^−/−^ mice do not alter IOP (Fig. 2), *Trpv4*^−/−^ eyes are likely to undergo compensatory upregulation of functionally cognate mechanisms (e.g., (49)). IOP increases observed in diurnal eyes injected with GSK101 and the significant IOP reductions induced by TRPV4 antagonists are consistent with hypertensive effects of TRPV4 activation.

### TRPV4 inhibition lowers IOP in the iridocorneal occlusion OHT

**Figure 3:**
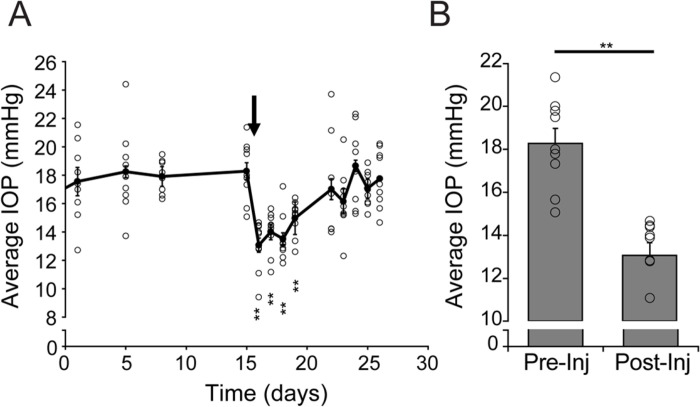
TRPV4 maintains diurnal in the microbead occlusion model of glaucoma. (**A**) Time course of average IOP stably elevated by intracameral MB microinjections. Following OHT induction ipsilateral eyes were injected intracamerally with 100 μM HC-06 (black arrow) and IOP measured daily for five days (N = 9 eyes). Error bars represent mean ± SEM. Significance for post-injection time points was calculated against the time point immediately preceding the MB injection. Repeated-measures one-way ANOVA with Dunnett’s post-hoc comparisons. **P < 0.01. (**B**) Comparison between the average pre-injection IOP from A (t = 15d) and the 24-hour time point after the injection. Data represent mean ± SEM. ** p < 0.01.

The IOP-lowering efficacy of actin depolymerizing agents and Rho kinase inhibitors in rodents and primates(13, 50, 51) links increased outflow resistance in hypertensive eyes to continual activity of the contractile apparatus downstream from pressure sensing. We tested the hypothesis that pressure-dependence of OHT requires TRPV4 in the microbead (MB) model that mimics pigmentary glaucoma in rodents(52) and non-human primates (53). Injections of magnetic polystyrene beads stably elevated IOP for several weeks (Fig. 3A and B). Intracameral injection of HC-06 during week two of OHT consistently lowered IOP by ~ 27%. The hypotensive effect of HC-06 was detected at the first time point of post-injection measurement (i.e., within 24 hours of antagonist exposure) and lasted between 4–5 days (Fig. 3A).

**Figure 4:**
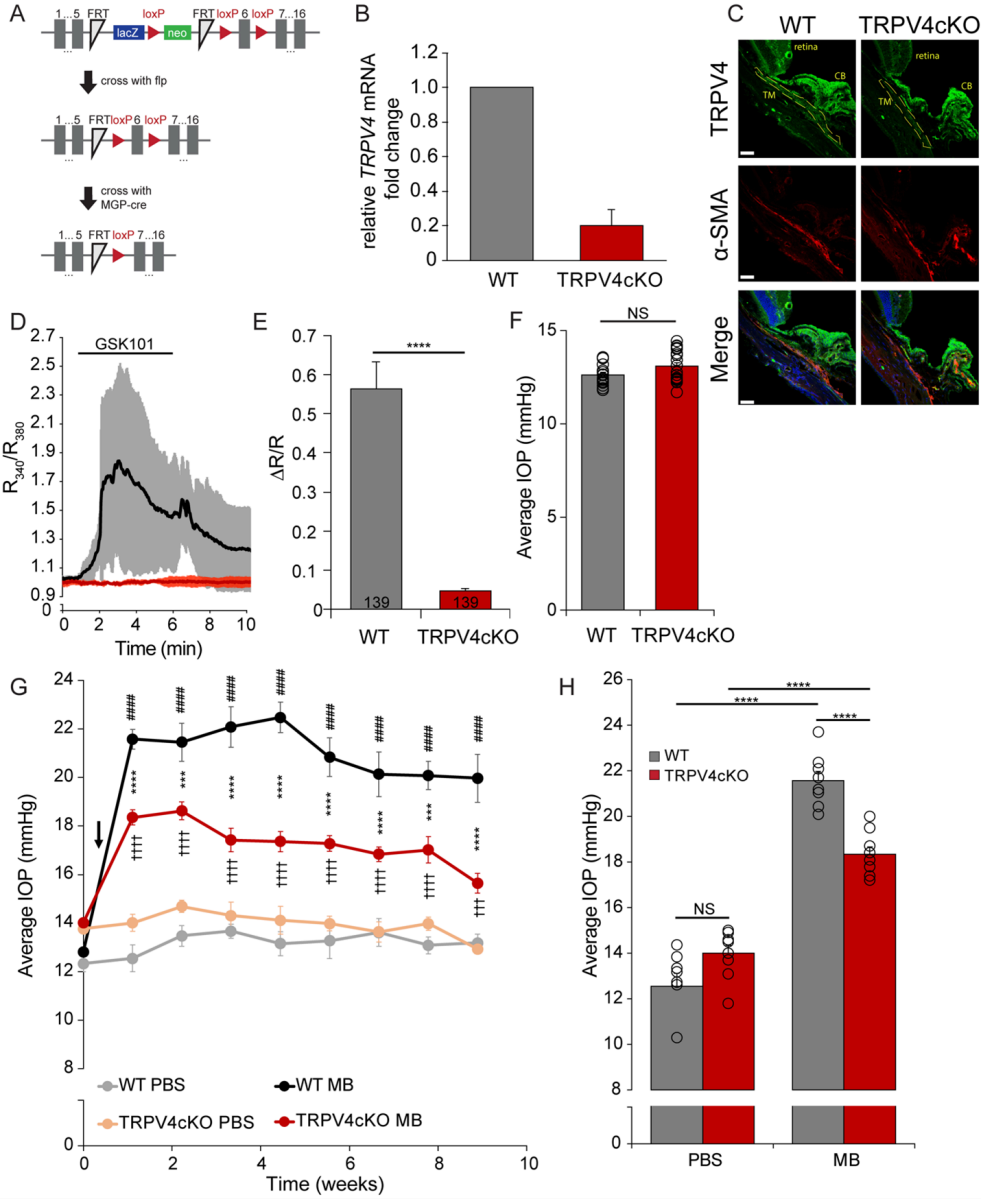
OHT is reduced in animals with TRPV4 conditionally ablated from the outflow pathway. (**A**) Gene targeting strategy for ablating TRPV4 from the conventional outflow pathway. (**B**) qPCR analysis for *Trpv4* mRNA in mTM cells isolated from WT and *Trpv4* cKO animals. (**C**) Immunohistochemistry in WT vs *Trpv4* cKO cryosections of the iridocorneal angle, stained for TRPV4 (green), alpha smooth muscle actin (aSMA, red) and DAPI (blue). TM is outlined in yellow dashed line in the TRPV4 panels. TM, trabecular meshwork, CB, ciliary body. Scale bar = 50 μm. (**D**) Representative time course of GSK101-evoked Ca^2+^ influx in WT (n = 4; black trace) and *Trpv4* cKO (n = 5; red trace) mTM cells exposed to five minutes of 25 nM GSK101. Data represents mean ± SEM. (**E**) Quantification of calcium imaging experiments in WT and *Trpv4* cKO mTM cells (N = 139 cells per group; data collated from three independent calcium imaging experiments), mean ± SEM. Statistical significance was calculated with a two-tailed t-test. ****P < 0.0001. (**F**) Diurnal IOP (mm Hg) in WT mice (N = 17) and *Trpv4* cKO mice (N = 21), calculated as the average IOP of two consecutive days prior to MB-OHT induction. Two-tailed t-test. (**G**) Average IOP over the course of 9 weeks in WT and *Trpv4* cKO mice injected with PBS vehicle (WT: N = 8 eyes, *Trpv4* cKO: N= 9 eyes) ipsilaterally and MBs (WT: N = 8 eyes, *Trpv4* cKO: N= 9 eyes) in the contralateral eye. Data, mean ± SEM. Asterisks represent statistical comparison between WT MB and *Trpv4* cKO MB, # represent comparisons between WT PBS and WT MB, † comparison between Trpv4 cKO PBS and Trpv4 cKO MB (Two-way ANOVA with Tukey multiple comparison). ***P < 0.001, ****P < 0.0001. (**H**) Quantification of data from G at 24 hours after the PBS/MB injection. Data represents mean ± SEM. NS = non-significant,

To determine whether the IOP-lowering effect of TRPV4 inhibition involves channels endogenous to the outflow pathway, we ablated the *Trpv4* gene using a mouse line with the Cre recombinase linked to the gene encoding the Matrix Gla (MGP) protein(54) *Mgp*^*Cre*^ mice were mated to *Trpv4*^*fl/fl*^ animals generated from ES clones obtained from the KOMP repository (*Trpv4*^*trn1a(KO<P)Wtsi*^) (Fig. 4A), with homozygous descendants showing loss of *Trpv4* expression within the TM (Fig. 4B) but not other TRPV4-expressing cells and tissues (55). No structural phenotype was apparent in TM layers or other tissues in the anterior cKO eye. Similar to the lack of IOP difference measured in wild type vs. global *Trpv4*^−/−^ mice (Figs. 1A), baseline IOP in *Mgp*^*Cre*^*Trpv4*^*fl/fl*^ mice lacking TRPV4 expression in cells within the conventional outflow was comparable to IOP in WT animals (Fig. 4F). Indicating effective knockdown, cKO retinas lacked TM TRPV4-ir but maintained it in the ciliary body (Figs. 4C). In contrast to TM cells isolated from wild-type animals, cKO TM cells failed to respond to GSK101 with [Ca^2+^]_i_ elevations (Fig. 4D and E). Interestingly, MB-induced OHT in cKO animals was significantly lower compared to OHT measured in wild type mice (Fig. 4G and H). TRPV4 channel activation thus appears to be required to sustain OHT but does not contribute to normotension.

### TRPV4 inhibition attenuates dexamethasone induced OHT

**Figure 5:**
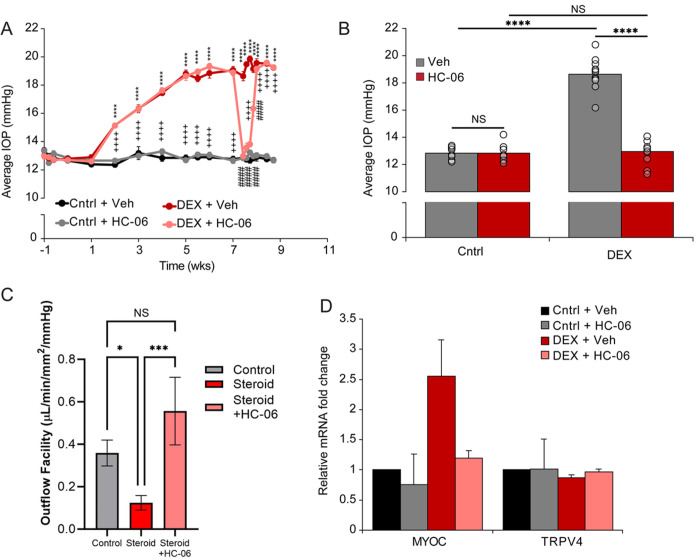
TRPV4 inhibition reduces IOP and increases outflow facility in the steroid-induced model of glaucoma. (**A**) Ocular hypertension was induced by 3x daily administration of DEX (0.1% ophthalmic solution) eye drops in both eyes of mice assigned to the DEX cohort (N = 10 mice). A subset of age-matched controls was treated with a sterile, control eye drop (N = 10). HC-06 (100 μM) or DMSO/PBS vehicle (Veh) were injected into the contralateral and ipsilateral anterior chambers, respectively of both control and DEX treated mice. After HC-06 microinjection IOP was measured every 24 hours for six consecutive days. Data represents mean ± SEM. Asterisks represent the statistical comparison between DEX + Veh and Cntrl + Veh; # statistical comparison between DEX + Veh and DEX + HC-06; † statistical comparison between DEX + HC-06 and Cntrl + HC-06 (two-way ANOVA with Tukey multiple comparisons). Comparisons not shown are not statistically significant. **** P < 0.0001. (**B**) Quantification of data from A 24 hours after HC-06 injection. N = 10 eyes per group. Data represents mean ± SEM. NS = not significant, **** P < 0.0001. (**C**) pTM cells were seeded onto nano-scaffolds and perfused at flow rates of 2,4, 8, 16 ml/min in the presence or absence of 9-PA. 9-PA (1%) decreases the outflow facility in 3D pTM cultures, an effect that is reversed by preincubation with 2 μM HC-06 (N = 6 scaffolds). Data represents mean ± SEM. Statistics were calculated with an ANOVA with Bonferroni post-test. NS = not significant, * P < 0.05, ***P < 0.001. (**D**) RT-PCR, pTM cells. Pre-incubation with HC-06 suppresses DEX-induced expression of MYOC; Trpv4 transcription is not affected by DEX or HC-06 exposure (N = 3).

To determine whether the TRPV4-dependence of OHT applies to IOP elevations induced by chronic glucocorticoid exposure, a prevalent health threat in human patients (56), IOP was stably elevated with 3x daily administration of dexamethasone (DEX). Reflecting the steroid-dependence of the facility (57), the average IOP in DEX-treated wild type mice was stably elevated within 5 weeks of chronic steroid treatment. HC-06 injection into OHT eyes produced consistent and reversible IOP lowering that lasted for several days (Fig. 5A and B). These results therefore indicate that persistent activation of TRPV4 is obligatory for tonic stimulation of the contractile apparatus that maintains OHT in steroid-treated animals (51).

To ascertain the TRPV4-dependence of outflow in steroid-treated TM cells in the absence of Schlemm’s canal, ciliary body, and ciliary muscle mechanisms, we employed a bioengineered 3D nanoscaffold system that has been used widely to investigate the role of HC-06 in facility modulation (23, 58, 59). TM cells were seeded onto micropatterned SU-8 scaffolds and placed in a perfusion apparatus that simulates ocular flow in the presence/absence of prednisolone acetate (PA), which we found previously suppresses the facility *in vitro* (60). The outflow facility in PA-treated preparations was significantly reduced compared to untreated controls, an effect that was obviated by HC-06 which produced ~ doubling of outflow facility the levels in untreated control preparations (Fig. 5C). These data indicate that glucocorticoid-induced increases in outflow resistance are partially mediated through TRPV4 activation and can be dynamically reversed through TRPV4 inhibition.

### Conditional ablation of TRPV4 channels is neuroprotective

**Figure 6:**
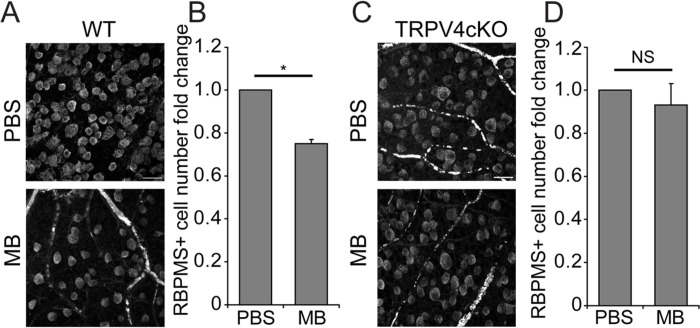
Ablation of TRPV4 from the conventional outflow pathway protects RGCs from pressure-induced injury. (**A**) Representative wholemount views of the RGCL immunolabeled for RBPMS in eyes injected with PBS or MB in WT mice. Scale bar = 20 μm (**B**) Quantification of data presented in A. Data represent the average of quantifications from four peripheral quadrants from each WT retina (N = 4 mice) normalized to the total number of RBPMS^+^ cells in the PBS treated eyes ± SEM. Two-tailed t-test, *P < 0.05 (**C**) Representative wholemount views of the RGCL immunolabeled for RBPMS in eyes injected with PBS or MB in *Trpv4* cKO mice. Scale bar = 20 μm (**D**) Quantification of data presented in C. Data represent the average of quantifications of four peripheral quadrants from each *Trpv4* cKO retina (N = 4 mice). Data represents mean ± SEM. Two-tailed t-test, NS = non-significant.

We studied the long-term significance of tonic TRPV4 signaling within the conventional pathway by assessing RGC viability in retinas from eyes that experienced three months of OHT. Consistent with previous reports (23, 61), MB-induced OHT in animals with iridocorneal occlusion was associated with ~ 25% loss of RBPMS^+^ cells in the peripheral retina (Fig. 6A and B). Remarkably, *Trpv4*^−/−^ cKO retinas were protected from OHT-dependent injury (~ 7% RBPMS^+^ cell loss), with RGC counts in the central retina of hypertensive cKO eyes not different from normotensive controls. Targeting TRPV4 channel expression and function within the anterior eye might therefore serve as a neuroprotective strategy in glaucoma.

## DISCUSSION

This study shows that TRPV4 channels are required for induction of OHT by the circadian cycle and pathological interventions that mimic primary and secondary glaucoma. Pharmacological approaches combined with facility analyses and IOP measurements in animals with globally and conditionally ablated TRPV4 gene indicate that (i) TRPV4 activity maintains OHT induced by nocturnal, occlusion and steroid-induced conditions; (ii) TRPV4 inhibition reverses the facility-suppressive effect of circadian rhythmicity, steroid exposure and angle occlusion, (iii) TRPV4 agonism affects IOP in an administration location-specific manner, (iv) Global KO animals experience compensatory changes in pressure sensing, and (v) TRPV4 knockdown from cells within the conventional outflow pathway is sufficient to phenocopy the effect of pharmacological inhibition. TRPV4 overactivation thus appears to function as an outflow brake that can be targeted in primary and secondary glaucoma cases that resist current treatments. The (vi) protection of RGCs in *Trpv4* cKO retinas from pressure-induced injury additionally implicates the channel in the progression of glaucomatous neurodegeneration.

Identification of the elusive transducer molecules that mediate homeostatic and pathological increases in conventional outflow resistance has represented a main quest in glaucoma research over the past fifty years (10, 62, 63). TM mechanotransduction underpins pressure-evoked changes in outflow resistance (64, 65) through a multi-stage process that consists of force-coupling by the sensor protein, force conversion into intracellular signals, and reorganization of signaling pathways that maintain ECM secretion and the contractile machinery. This study shows that the TRPV4 channel, previously shown to mediate the principal component of the pressure-activated transmembrane current in primary and immortalized human TM cells (20) and subserve TM responsiveness to mechanical strain (23), is obligatory for the induction and maintenance of OHT and establishes a mechanistic framework that accounts for the Ca^2+^-dependence of TM fibrosis, Rho signaling and contractility that represent the molecular, and druggable, foundation of OHT. Specifically, we report that: (i) TRPV4 antagonists lower IOP at night-time and in MB- and DEX-treated eyes; (ii) TRPV4 antagonists augment the outflow facility by alleviating the suppressive effects of steroids (Fig. 5) and mechanical stress (23) by acting on the TM in the absence of inflow and secondary outflow mechanisms; (iii) Selective ablation of TRPV4 channels from the outflow pathway reduces OHT amplitude and protects RGCs from IOP-induced degeneration, (iv) IOP is elevated by intracameral delivery of GSK101 and lowered by topical administration of the agonist, and (v) pharmacological inhibition, global and conditional TRPV4 ablation have no effect on diurnal normotension. Our central observation is that conditional ablation of TRPV4 channels and microinjections of TRPV4 antagonists reduce OHT under physiological and pathological conditions. HC-06, considered a selective TRPV4 antagonist with few if any, nonspecific targets (66), eliminated the slow pressure-evoked current *in vitro* (20), reduced the amplitude of stretch-evoked [Ca^2+^]_i_ responses (23) and induced powerful and long-lasting IOP reductions in nocturnal and MB-induced OHT paradigms (Figs. 1–5). We controlled for potential off-target effects with a structurally distinct antagonist (GSK219), by testing the effects of the agonist (GSK101), by conditionally ablating the TRPV4 gene from the conventional pathway, by testing global *Trpv4*^−/−^ mice, and by establishing the TRPV4-dependence of the outflow facility in an *in vitro* biomimetic outflow system in the absence of inflow, Schlemm’s canal and uveoscleral components. The Humonix technique, consisting of a porous nanoscaffold seeded with human TM cells, has been widely used to define the hydraulic conductivity of trabecular outflow under many glaucoma-relevant conditions (23, 58, 60, 67–69). Consistent with previous work (60, 69) we found that TM-populated nanoscaffolds respond to glucocorticoids with facility lowering. TRPV4 inhibition not only rescued the suppressive effect of the steroid but elevated facility ~ twofold above its control value. These findings corroborate the HC-06 -dependence of facility reported previously (23) and expand the mechanistic context for the dramatic increase in outflow resistance seen in GSK101-treated preparations (23). Mechanistically, the GSK101-dependence of outflow resistance corresponds the IOP increase induced by intracameral delivery of the agonist (Fig. 1D).

TRPV4 is a polymodal cation channel that mediates the principal component of the pressure-evoked cation current (I_pressure_) (20), underpins TM responsiveness to physiological (3–12%) stretch (23, 25), swelling (70), pressure(20, 71) and shear (24), and can be activated by PUFA metabolites arachidonic acid and 5’6’-EET and small molecule agonists GSK101 and 4a-PDD (22, 23). Despite its clear role in TM pressure transduction and stretch-induced Ca^2+^ homeostasis, the functional significance of TRPV4 activity has been debated in studies that linked it to IOP lowering(22, 24, 25) vs. elevation (23). In addition, TRPV4 mechanosensing has been associated with a multiplicity of signaling pathways that include phosphoinositide metabolism within primary cilia (22), nitric oxide release (24, 25), PUFA release (25), RhoA-ROCK signaling (45), lipid remodeling(72) and Piezo1 activation (73). Unfortunately, experimental evidence seems inconsistent with some of these mechanisms as ablation of TM primary cilia has no effect on TRPV4 signaling (23), eNOS expression in mammalian TM cells is vanishingly low or absent (24, 74, 75), phospholipase A2 metabolites such as such as arachidonic acid activate rather than inhibit, TRPV4(23) and TRPV4 activation is unaffected by Piezo1 inhibition (20). In agreement with Patel et al. (24), we found that eye drops of GSK101 induce significant IOP lowering when applied during the hypertensive nocturnal phase (Fig. 1) yet also observed that the agonist elevates IOP when its accessibility is optimized through intracameral delivery. These observations narrow the spectrum of likely signaling pathways downstream from TRPV4-mediated Ca^2+^ influx and point at Ca^2+^-dependent actomyosin contractility the most likely common final mechanism of TRPV4 activation. Consistent with this conjecture, GSK101 stimulation upregulates formation of stress fibers by driving the TRPV4-RhoA-ROCK axis (23, 45), and stimulates fibronectin release to directly suppress trabecular outflow (23). Other outflow-suppressive effects of TRPV4 activation include tyrosine phosphorylations of RhoA and key focal adhesion proteins (e.g., paxillin, focal adhesion kinase, vinculin), polymerization of F-actin, ROCK activation, increased aSMA expression, translocation of phosphorylated zyxin into stress fibers, reinforcement of focal cell-ECM contacts and release of ECM proteins (23, 45). RhoA constitutes a quasi-subunit of TRPV4 that dissociates from the protein in response to Ca^2+^ influx to bind its effector ROCK (48, 76, 77). Similar to our results, investigations of TRPV4 signaling in fibroblasts, smooth muscle, immune and epithelial cells and fluid-regulating organs (cardiovascular system, brain, kidney, and joint cavities) link Ca^2+^ influx to RhoA-ROCK activation and assembly of actin/aSMA (78). Conversely, TRPV4 ablation results in dephosphorylation of RhoA, suppression of actin polymerization, and attenuated contractility(79, 80) and removal of extracellular calcium reduces aSMA incorporation into stress fibers and prevents transdifferentiation into contractile myofibroblasts (80). Given that IOP lowering in eyes treated TRPV4 blockers mirrors the effect of actin depolymerizing drugs and ROCK inhibitors, we propose that TRPV4 blockers/ablation, ROCK inhibitors, and latrunculins/cytochalasin D target different stages of the same mechanosensitive pathway.

We propose that topically applied GSK101induces hypotension by stimulating release of outflow-promoting factors from TRPV4-expressing cornea, ciliary body, ciliary muscle and/or sclera (39). We and others showed that corneal and ciliary body epithelia respond to the agonist with massive Ca^2+^ waves and hemichannel-mediated release of ATP (81–83), with ATP, its purine and adenosine derivatives and downstream effectors (e.g., matrix metalloproteinases) known to lower IOP by acting on P2 receptors, ATP-sensitive K^+^ channels, adenosine A1 receptors and MMP-dependent ECM remodeling (84–88). The TRPV4-dependence of IOP homeostasis was unmasked by pharmacological inhibition, which induced 80–95% hypotension in eyes under nocturnal, microbead- and steroid-treated conditions. Conditional ablation of the channel from the outflow pathway similarly produced ~ 50% lowering of OHT, with the difference between pharmacological vs. genetic paradigm potentially reflecting incomplete knockdown, upregulation of cognate mechanisms and/or involvement of inflow and uveoscleral mechanisms. Mechanosensitive, TRPV4-ir lympho-endothelial cells that form the canal of Schlemm(11, 23, 63, 89) might, for example, oppose the outflow-suppressive effects of TM-resident TRPV4 channels. TRPV4 agonists tend to dilate vascular and lymphatic vessels due to Ca^2+^-dependent NO release, K^+^ channel activation and dissolution of cell-cell junctions (36, 90, 91). We found, for example, that GSK101 promotes vascular permeability (36) and downregulation of endothelial TRPV4 channels has been generally observed to suppress vascular relaxation (92, 93). Another potential effector of in vivo IOP responses to TRPV4 modulation might be the ciliary body, which expresses TRPV4 within the nonpigmented epithelium(83) where it could be associated with volume regulation and/or aqueous humor secretion (31, 94, 95). Our observations that (i) GSK101 reduces, and HC-06 stimulates, outflow in biomimetic scaffolds that lack inflow, uveoscleral and SC components, that these effects (ii) accord with IOP increases following GSK101 microinjection and IOP decreases following HC-06 microinjection, and that (iii) TRPV4 ablation from the outflow pathway largely phenocopies the effect of TRPV4 inhibition, argue major roles of inflow and uveoscleral mechanisms in HC-06- and KO/cKO-dependent IOP lowering.

The TRPV4-dependence of the remarkable facility increases in glucocorticoid-treated cells (Figs. 5 and 6) implicates the channel in outflow suppression in steroid glaucoma. Glucocorticoid implants and eye drops are often prescribed to treat macular edema, corneal transplantation and injury-related inflammation but constitute a double-edged sword, with 30–40% of the patients (‘steroid responders’) and 90% of POAG patients developing OHT (96–98). GSK101, mechanical strain and steroid treatments steer TM cells toward the contractile myofibroblast phenotype (99, 100) that may rescued by targeting the mechanotransducer-Rho signaling axis, as indicated by OHT reversal in DEX-treated patients (101) and mice (51) treated with ROCK and TRPV4 inhibitors. Studies in epithelial cells and neurons indeed implicate glucocorticoid exposure in increased TRPV4 gene expression, trafficking and/or lowering of mechanical thresholds (102–105). Future studies will dissect the TRPV4-dependence of OHT in animal models together with delineation of molecular pathways associated with GCR/Nr3C1 signaling.

A corollary of the present study is that TRPV4 may be required for the circadian IOP shift in addition to subserving pathological OHT induction. This possibility is based on the IOP lowering induced by HC-06 microinjections during the nocturnal hypertensive phase of the circadian cycle, in MB-treated and steroid-treated mice. Nocturnal TRPV4 activation is likely to be associated with Rho signaling yet uncoupled from Ca^2+^-dependent fibrotic programs, as suggested in our studies in which a single dose of the dominant negative RhoA vector (*scAAV2.dnRhoA*) sufficed to block nocturnal OHT without affecting TM and SC morphology (54). The nonfibrotic signaling that underlies the circadian IOP switch(106) is presumably under the control of the paraventricular nucleus, suprachiasmatic nucleus (SCN) and the hypothalamic ventral tuberomammillary nucleus (VTM) and may involve activation of *Cry1,2* cryptochrome, *Clock* gene and calcium (phosphoinositol-3,4,5-trisphosphate; PIP_3_) pathways (47, 107), with the Rho-dependence of fibrotic differentiation attenuated by upregulated melatonin signaling (108, 109).

The comparable range of normotensive IOP values in diurnal wild type, global *Trpv4*^−/−^ and *Mgp*:cKO animals argues against major functions for TRPV4 activity in maintaining balanced AH inflow *vs*. outflow. Our data are instead consistent with activation of the channel by increases in ECM strain, which destabilize the tensional steady-state to trigger TRPV4-mediated Ca^2+^ influx and attendant gene expression, cytoskeletal and cell-ECM remodeling mechanisms (23, 45). Chronic overactivation of this mechanism leads to TM pathology that mirrors mechanically induced remodeling across the body, as indicated by inhibited ECM remodeling, aSMA overexpression and myofibroblast transdifferentiation that follow TRPV4 inhibition and knockdown *(*110 *−* 113*)*. It remains to be seen whether normotension relies on TM-endogenous TREK-1 and Piezo1 channels(19–21) and/or involves TRPM4 channels (70, 71). The reduction in*ex vivo* and *in vivo* facility (20, 21) in eyes perfused with Piezo1 antagonists implicates this fast-inactivating channel in transient facilitation of homeostatic outflow. The role of TREK-1 in is less well understood, with TREK-1 agonists shown to augment the permeability of TM monolayers(19) yet *Kcnk2* knockdown associated with OHT (18). Also worth noting is the extraordinary context-dependence of TRPV4 signaling that is subserved by many accessory and adaptor proteins (26), the important role of TRPV4-lipid interactions (e.g., PIP2 binding and cholesterol-phospholipid-TRPV4 interactions) (114), integrin and focal adhesion signaling (27, 45, 115), and potential functions of TRPV4 signaling for exosome release and transcription.

In conclusion, this study provides evidence that TRPV4-mediated pressure transduction subserves mechanically- and steroid-induced outflow remodeling as the final common mechanism that can be targeted to mitigate OHT. The pathobiological relevance of TRPV4 signaling is accentuated by the protection of retinal neurons from pressure-induced injury in animals with conditionally ablated TRPV4 channels. MB-treated cKO eyes showed ~ 50% reduction in IOP together with significant neuroprotection (Figs. 4 and 6), indicating that the residual mechanical stress is insufficient to cross RGC viability mechanothresholds. Similar observations in patients associated ~ 20% IOP decrease with substantial alleviation of glaucoma risk (116). *Trpv4*^−/−^ eyes show normal anatomy of the anterior chamber, circadian IOP rhythmicity and light-evoked signaling (23, 117), suggesting that targeting the channel can obviate ocular hyperemia without major structural or functional side effects (118, 119). Because TRPV4 tends to be localized to cell types that are susceptible to glaucoma (RGCs, Müller glia, microglia, endothelial cells) (120–123), its targeting may achieve the quadfecta of IOP lowering, reduced neuroinflammation, reduced ischemia, and neuroprotection.

## Figures and Tables

**Table 1:** 

Name	Forward primer	Reverse primer	NCBLNM
***CHI3H1***	GACCCTGGCCTACTACGAGA	ATTCCTTCGGCTGACAGGTG	007695.4
***FN1***	CCCTCCATCTTTGAGTGGTCC	AACCCTGAAGCAGAACAGGG	010233.2
***MGP***	CAACAAGCCTGCCTACGAGA	AAGGAGCAAGCAACACATGC	008597.4
***TIMP3***	TTCTGTCTCCCAACCAAGGA	CCAACTACCTGAATGGCACCT	011595.2
***MMP2***	GGTGTGCCAAGGTGGAAATC	AGGTTGAAGGAAACGAGCGA	008610.3
***TRPV4***	TCCTGAGGCCGAGAAGTACA	TCCCCCTCAAACAGATTGGC	022017.3
***MYOC***	CCACGTGGAGAATCGACACA	TCCAGTGGCCTAGGCAGTAT	000261.1
***Gapdh***	GGTTGTCTCCTGCGACTTCA	TAGGGCCTCTCTTCCTCAGT	001289726.2
